# Machine Learning-Based Prediction of Prostate Biopsy Necessity Using PSA, MRI, and Hematologic Parameters

**DOI:** 10.3390/jcm14010183

**Published:** 2024-12-31

**Authors:** Mustafa Sungur, Aykut Aykaç, Mehmet Erhan Aydin, Ozer Celik, Coskun Kaya

**Affiliations:** 1 Department of Urology, Health Science University Eskisehir City Health Application and Research Center, 26080 Eskisehir, Turkey; aykutdr@gmail.com (A.A.); mehmeterhan.aydin@saglik.gov.tr (M.E.A.); coskun.kaya@sbu.edu.tr (C.K.); 2 Department of Mathematics and Computer, Faculty of Science, Eskisehir Osmangazi University, 26130 Eskisehir, Turkey; ozer@ogu.edu.tr

**Keywords:** prostate biopsy, prostate cancer, machine learning, artificial intelligence

## Abstract

**Background:** To establish a machine learning (ML) model for predicting prostate biopsy outcomes using prostate-specific antigen (PSA) values, multiparametric magnetic resonance imaging (mpMRI) findings, and hematologic parameters. **Methods:** The medical records of the patients who had undergone a prostate biopsy were evaluated. Laboratory findings, mpMRI findings, and prostate biopsy results were collected. Patients with benign prostate pathology were classified as Group 1, and those with prostate cancer (PCa) were classified as Group 2. The following ML algorithms were used to create the ML model: ExtraTrees classifier, Light Gradient-Boosting Machine (LGBM) classifier, eXtreme Gradient Boosting (XGB) classifier, Logistic Regression, and Random Forest classifier. **Results:** A total of 244 male patients who met the inclusion criteria were included in this study. Among them, 171 (71.1%) were categorized in Group 1, and 73 (29.9%) in Group 2. The LGBM classifier model demonstrated the highest performance, achieving an accuracy rate of 81.6% and an AUC–ROC (area under the curve–receiver operating characteristic) of 78.4%, with sensitivity and specificity values of 66.7% and 88.2%, respectively, in predicting prostate biopsy outcomes. **Conclusions:** Pathological results can be predicted by ML models using PSA values, mpMRI findings, and hematologic parameters prior to a prostate biopsy, potentially reducing unnecessary biopsy procedures.

## 1. Introduction

Prostate cancer (PCa) poses a significant health risk to men, severely impacting both quality of life and life expectancy. The prostate-specific antigen (PSA) test is widely used in PCa diagnosis, as elevated PSA levels can indicate cancer risk. Early diagnosis and treatment are crucial to mitigate the adverse effects associated with the disease. A prostate biopsy remains a cornerstone diagnostic tool for confirming the presence of PCa. However, the specificity of PSA testing is limited. PSA levels can also rise due to benign conditions, such as prostate enlargement or inflammation, leading to unnecessary biopsies. PSA has been shown to be affected by certain conditions, even in PCa patients. Smokers with PCa tend to show higher PSA levels at the time of the radical prostatectomy. There are also different results in the literature regarding the effect of body mass index, history of chronic obstructive pulmonary disease, or heavy drinking on PSA [1]. Despite the routine use of biopsy in urological practice, it is not without risks, including potential mortality. Additionally, its diagnostic accuracy depends on the presence of malignant cells in the sampled tissue, which can lead to false negatives in clinically suspected cases. In response to these challenges, novel diagnostic techniques have been developed to enhance diagnostic accuracy and reduce the frequency of unnecessary biopsies. mRNA-based screening tests, including the Prostate Health Index, 4K score, IsoPSA, PCA3, SelectMDx, My Prostate Score (MPS), and ExoDx, have been introduced as potential diagnostic tools [2]. However, the high financial cost and suboptimal accuracy of these tests have limited their widespread adoption.

Recent studies have indicated that approximately 30% of patients categorized as low risk based on multiparametric prostate magnetic resonance imaging (mpMRI) may avoid unnecessary biopsy procedures. Nevertheless, inconsistencies in mpMRI reporting and the failure to detect approximately 15% of clinically significant PCa cases have prevented these techniques from achieving the desired outcomes [3].

To better identify individuals who genuinely require a biopsy, various prediction tools, and risk calculators have been developed. These tools incorporate PSA values and kinetics, mpMRI findings, digital rectal examination results, and other sociodemographic variables. Despite these efforts, the predictive accuracy of these tools remains suboptimal, and only a few have undergone external validation [4,5].

Hematologic parameters, such as the neutrophil-to-lymphocyte ratio (NLR), lymphocyte-to-monocyte ratio (LMR), and systemic immune-inflammation index (SII), have gained attention as potential biomarkers reflecting systemic inflammatory responses associated with malignancy [6,7,8].

The hemoglobin, albumin, lymphocyte, and platelet (HALP) score, which consists of the hemoglobin, albumin, lymphocytes, and platelet levels, to predict PCa has also been studied. These markers are indicators of nutrition status, and the immune system seems to be associated with PCa. These markers, when combined with PSA values and imaging findings, could provide a more comprehensive and precise approach to risk stratification, reducing unnecessary biopsies and associated complications [9].

In this context, innovative approaches, such as machine learning (ML) and artificial intelligence (AI) models, have significant potential in PCa diagnosis due to their ability to uncover complex patterns within datasets [5]. ML represents a paradigm shift in the diagnostic landscape by offering advanced computational techniques capable of analyzing large, high-dimensional datasets. Unlike traditional methods, ML algorithms can identify intricate patterns and non-linear relationships among variables, making them particularly suited for medical diagnostics. In the context of PCa, ML has been applied to various tasks, including biopsy outcome prediction, tumor grading, and treatment response assessment. However, despite the growing body of evidence supporting ML’s potential, and that there has been a surge in research focused on the diagnosis, prognosis, and outcome prediction of PCa, driven by advancements in statistical techniques and AI [10], the literature remains sparse regarding the use of AI-based models for identifying individuals who require a prostate biopsy, and there remains a scarcity of studies integrating PSA values, mpMRI findings, and hematologic parameters to predict the necessity of prostate biopsy.

This study aims to develop an ML model that can predict the necessity of prostate biopsy by incorporating PSA values, mpMRI findings, and hematologic parameters, thereby potentially reducing unnecessary biopsies and associated complications.

## 2. Materials and Methods

### 2.1. Data Source and Study Workflow

**Figure 1:**
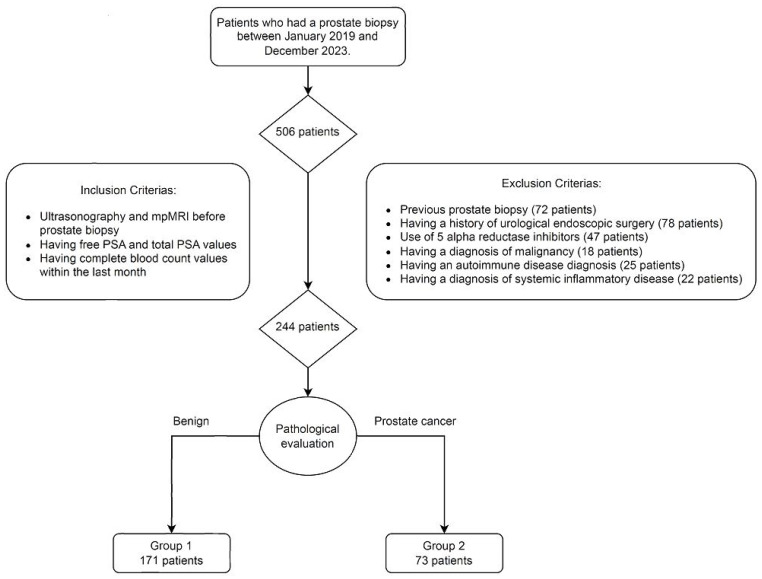
Flow Chart of this Study.

Following ethical approval (Eskisehir City Hospital, Non-Interventional Clinical Research Ethics Committee; Date: 16 February 2024; Decision Number: ESH/GOEK 2024/76)., we retrospectively evaluated Turkish patients who had undergone a prostate biopsy at the Eskisehir City Hospital between January 2019 and December 2023. The inclusion criteria required that patients had received a urinary ultrasonography and mpMRI prior to the biopsy, and had recorded free PSA, total PSA, and complete blood count values within the month before their biopsy procedure. Patients with previous prostate biopsies, a history of urological endoscopic surgery, use of 5-alpha-reductase inhibitors, a diagnosis of malignancy, autoimmune diseases, or systemic illness (like diabetes, cardiovascular diseases, chronic obstructive pulmonary disease, or obesity) were excluded from this study. The final cohort consisted of 244 patients (Figure 1).

### 2.2. Data Preprocessing

The patient data were recorded, including age, prostate volume, mpMRI PI-RADS score, free PSA, total PSA, neutrophil, lymphocyte, monocyte, and platelet counts, as well as the pathological evaluations. Calculated metrics included the free/total PSA ratio, PSA density (total PSA/prostate volume), NLR, LMR, platelet-to-lymphocyte ratio (PLR), and the SII, defined as neutrophils × platelets/lymphocytes. For patients with PI-RADS scores of 1 and 2 on their mpMRI, a standard 12-quadrant transrectal prostate biopsy was performed under local anesthesia. Cognitive fusion biopsy was additionally conducted for patients with PI-RADS scores of 3, 4, and 5. Based on the pathological evaluations, patients were categorized into two groups: those with benign prostate biopsy results and those with confirmed PCa. Missing data were managed systematically to minimize bias and maintain the integrity of the dataset. For cases where key predictor variables, such as PSA levels or hematologic parameters, were missing, listwise deletion was applied if the missing values exceeded a threshold of 20%. For missing values below this threshold, imputation methods, such as mean or median imputation, were used based on the variable distribution. If outcome data (prostate biopsy results) were missing, these cases were excluded from the analysis, as outcome data were essential for model training and validation. This approach ensured that the ML models were trained on a consistent dataset with complete outcome information, reducing the risk of introducing bias due to incomplete outcome data.

The normality of variables was assessed using the Shapiro–Wilk test. Continuous variables were expressed as mean ± standard deviation or median (range), while categorical variables were presented as frequency (percentage). Comparisons between groups for continuous variables with non-normal distributions were conducted using the Mann–Whitney U test, and associations between categorical variables were evaluated using the chi-squared test. A two-sided *p*-value < 0.05 was considered statistically significant in all analyses.

### 2.3. Machine Learning Models

Five ML algorithms were evaluated to achieve a robust prediction model: ExtraTrees classifier, Light Gradient-Boosting Machine (LGBM) classifier, eXtreme Gradient Boosting (XGB) classifier, Logistic Regression, and Random Forest classifier. These models were selected based on their diverse learning approaches and established performance in previous clinical predictive modeling [11].

Extra trees and random forest: These are ensemble methods that leverage multiple decision trees providing high performance in handling non-linear relationships and managing high-dimensional data. Their robustness against overfitting is particularly beneficial in retrospective studies.

LGBM and XGB classifiers: These gradient boosting algorithms are known for their efficiency in training on large datasets and their strong predictive accuracy in structured data contexts. They are commonly applied in medical studies due to their ability to capture complex feature interactions.

Logistic regression: As a classical statistical model, logistic regression offers interpretability and serves as a benchmark that enables comparison with more complex ML models.

### 2.4. Performance Evaluation

Comparing these algorithms allows for the identification of the model that best captures the relationships between PSA values, mpMRI findings, and hematologic parameters in predicting prostate biopsy outcomes. By assessing a variety of ML approaches, we aimed to ensure a balanced evaluation that could identify the optimal model in terms of accuracy, sensitivity, and specificity. The area under the curve (AUC) from the receiver operating characteristic (ROC) curve analysis was also calculated to evaluate the performance of the 5 models. For each ML model, 80% of the dataset was reserved for training and the remaining 20% for testing. Cross-validation, a statistical method widely used in applied ML, was employed to compare the results of the different models and to estimate performance.

### 2.5. Feature Selection

**Figure 2:**
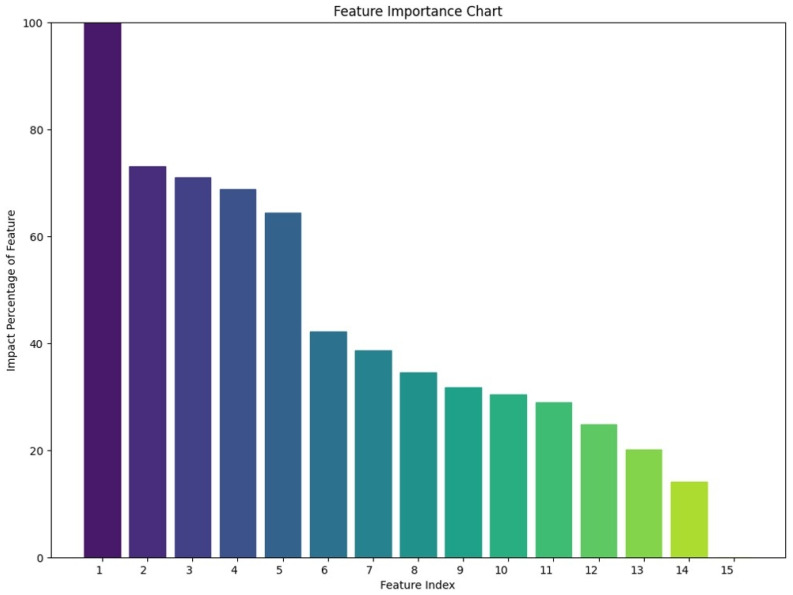
Feature Importance Chart of the Variables (1: Free/Total PSA, 2: Age, 3: Platelet, 4: PSA Density (Total PSA/Prostate Volume), 5: Total PSA, 6: PI-RADS Score, 7: Prostate Volume, 8: Lymphocyte-to-Monocyte Ratio, 9: Free PSA, 10: Systemic Immune Inflammation Index (Neutrophil × Platelet/Lymphocyte), 11: Neutrophil, 12: Neutrophil-to-Lymphocyte Ratio, 13: Lymphocyte, 14: Platelet-to-Lymphocyte Ratio, 15: Monocyte).

The permutation feature importance method, a robust approach for evaluating the contribution of individual features to the model’s performance, was employed to identify the most influential variables in this study. This method quantifies feature importance by assessing the reduction in the model’s predictive accuracy when the values of a single variable are randomly shuffled, thereby disrupting its relationship with the target variable. By systematically isolating each feature’s impact, this technique ensures an unbiased and comprehensive evaluation of their predictive value. The identified key features, ranked by their relative importance, are visualized in the Permutation Feature Importance Plot presented in Figure 2.

All statistical analyses were performed using IBM SPSS for Windows version 15.0, while ML algorithm tests were conducted with Python software (Python Software Foundation, Python Language Reference version 3.5).

## 3. Results

Of the 506 patients who had undergone a prostate biopsy at the urology clinic of Eskisehir City Hospital between January 2019 and December 2023, 244 met the inclusion criteria for this study. From post-pathological evaluation, 171 patients (71.1%) with benign biopsy results were categorized in Group 1, and 73 patients (29.9%) with PCa were categorized in Group 2 (Figure 1).

The comparative analysis revealed that Group 2 exhibited an older age (*p* = 0.004), lower prostate volume (*p* < 0.001), higher PI-RADS scores (*p* < 0.001), elevated total PSA (*p* = 0.001), reduced Free/Total PSA ratio (*p* < 0.001), and increased PSA density (*p* < 0.001) compared to Group 1. Additionally, monocyte counts were higher in Group 2 (*p* = 0.010) (Table 1).

The permutation feature importance analysis revealed that free/total PSA, age, and platelet count were the three most influential variables, followed by PSA density and total PSA (Figure 2). These findings highlight the critical role of PSA metrics, patient demographics, and hematologic parameters in driving the model’s predictive performance.

**Figure 3:**
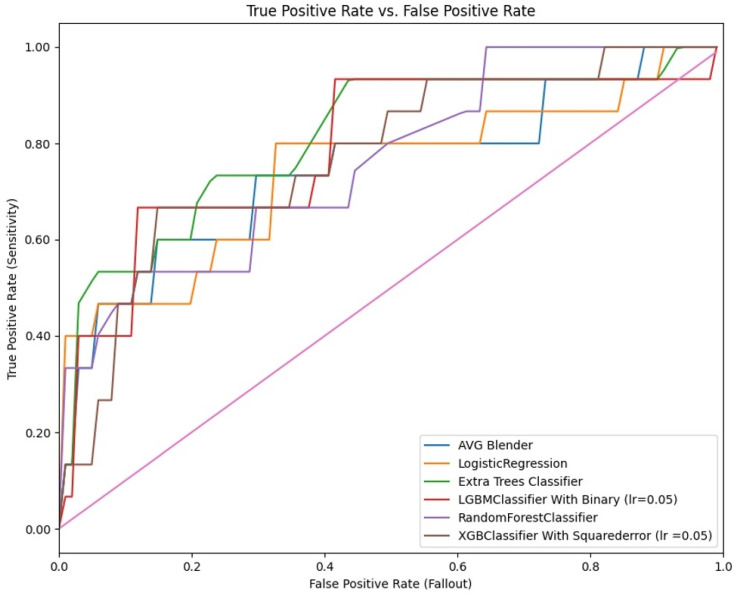
Receiver Operating Characteristic Curve Graphs.

Upon applying the ML algorithms to the test dataset, which represented 20% of the total data to evaluate the model’s performance, the LGBM Classifier demonstrated the highest predictive accuracy. Specifically, it achieved an accuracy rate of 81.6%, a sensitivity of 66.7%, a specificity of 88.2%, and an AUC–ROC of 78.4%. These results demonstrate the model’s capability to effectively distinguish between benign and malignant cases, reinforcing its potential use in reducing unnecessary prostate biopsies while maintaining high diagnostic precision (Table 2 and Table 3, Figure 3).

## 4. Discussion

Historically, the need for a prostate biopsy was determined by PSA levels and rectal examination findings. Although mpMRI has become standard practice in PCa detection, a consensus on the optimal approach to avoid unnecessary biopsies remains elusive. Traditional risk prediction tools, based on classical statistical methods, have limitations, including inaccuracies and insufficient integration into routine urological practice [2]. The focus on PCa has shifted to the “when and if” of treatment rather than the “how”. In this evolving landscape, the approach to prostate PCa therapy is shifting towards patient-specific strategies. Molecular and genetic analyses of tumor tissue, in conjunction with clinical risk factors, seek to provide a more profound understanding of disease aggressiveness. Several tissue-based prognostic markers have been developed for clinical use and are now commercially available. The Oncotype^®^ DX Genomic Prostate Score (GPS), the Prolaris^®^, and the ProMark^®^ have been designed to enhance tumor characterization and bridge the gap between clinical guidelines and actual clinical practice [12].

Previous studies have explored the potential of ML models to reduce unnecessary prostate biopsies by leveraging their ability to elucidate complex, high-dimensional relationships among various predictive factors [13,14,15,16,17,18,19]. For example, Suh et al. were the first to report on AI-based models for predicting prostate biopsy outcomes, achieving an AUC of 0.869 using variables such as total and free PSA levels, age, prostate volume, and hypoechoic lesions on ultrasonography [13]. Yu et al. compared ML methods with classical statistical approaches and found that ML models (support vector machine and random forest) underperformed compared to logistic regression [14]. Checcucci et al. developed a fuzzy classifier model incorporating PSA levels, mpMRI findings, and PI-RADS scores, which demonstrated predictive success in almost all cases [15,16]. The construction methods and performance parameters of these ML models were not consistently reported, limiting their reproducibility and application.

Chen et al. constructed a predictive model with ML techniques, incorporating hematologic parameters for the first time in the literature, and reported superior AUC and accuracy rates [18]. The recently introduced Prostate Cancer Artificial Intelligence Diagnostic System (PCAIDS) further enhanced diagnostic efficacy by utilizing 36 features selected by the random forest algorithm, though the complexity of these features may limit their routine clinical use [19].

In this study, we aimed to develop a robust ML model capable of predicting the necessity of prostate biopsy by integrating prostate-specific antigen (PSA) values, mpMRI findings, and hematologic parameters as there is increasing evidence that the inflammatory response may play a major role in the onset and development of various cancers. Studies have shown that inflammation-related neutrophils and immune cells, including lymphocytes, are indispensable participants in tumorigenesis. Among these inflammatory parameters, which are reflections of the systemic inflammatory response, an elevated NLR has been shown to be a valuable predictor of the clinical outcome in cancer patients. Furthermore, NLR is an easily available and reliable marker as it can be obtained from a routine blood test. In several malignant tumors, including PCa, a large number of studies have shown an association between elevated pre-treatment NLR and poor prognosis. With the heterogeneous character of PCa, none of the biomarkers, screening tests, or scoring systems, can provide diagnostic value, but by combining prostate imaging and tissue sampling they can be used together to provide a whole solution [9,20].

By combining the results of these diverse yet clinically relevant tests and systems, our model seeks to provide a comprehensive risk stratification tool that can accurately distinguish between patients requiring a biopsy and those who do not need it. PSA levels and their derivatives, such as PSA density and free-to-total PSA ratio, are widely recognized markers in PCa screening but are often insufficiently specific when used alone. The inclusion of mpMRI findings enhances the ability to localize and characterize suspicious lesions, offering anatomical and functional insights that complement PSA data. Furthermore, hematologic parameters, such as NLR, LMR, and SII, add another dimension by reflecting systemic inflammatory and immune responses, which are increasingly implicated in cancer progression and tumor microenvironment dynamics.

By leveraging the strengths of these data sources, the ML model aims to address critical limitations in current diagnostic workflows, including the over-reliance on only PSA and the subjective variability in mpMRI interpretations. This approach has the potential to significantly reduce unnecessary biopsies, thereby sparing patients from the associated risks, such as infection, bleeding, and psychological distress. Additionally, minimizing invasive procedures can alleviate the burden on healthcare systems by optimizing resource utilization and prioritizing biopsies for patients at the highest risk of clinically significant PCa.

The ultimate goal of this study is not only to improve diagnostic precision but to pave the way for integrating advanced computational tools into routine clinical practice. A successful ML model has the potential to serve as a decision-support system, empowering clinicians with evidence-based insights and enabling a more personalized approach to PCa management. This innovative framework aligns with the broader objectives of precision medicine, emphasizing tailored interventions and improved patient outcomes while reducing the overall impact of unnecessary medical procedures.

Our study represents the third instance in the literature where hematologic parameters and their dynamics have been used as features in ML models for PCa prediction. In this study, our primary aim was to accurately predict prostate biopsy outcomes using a combination of PSA values, mpMRI findings, and hematologic parameters. We selected PSA values, mpMRI (PI-RADS scores), and hematologic markers based on their established relevance in PCa diagnostics and their potential to improve predictive accuracy when used in combination. Our selection of algorithms, including the LGBM classifier and others, was informed by their proven effectiveness in handling structured data and delivering high accuracy. Each algorithm’s use is aligned with our aim to identify patterns and interactions that traditional methods might overlook. By comparing five widely used ML methods, we found that the LGBM classifier model outperformed others, particularly in terms of accuracy.

The strengths of our study include the selection of easily obtainable variables from routine urological practice, the description of the ML model construction process, and the comprehensive reporting of performance parameters. Nonetheless, our study has limitations, including its retrospective, single-center design, the inclusion of only Turkish patients, and the lack of external validation. Additionally, biopsies were conducted as 12-core systematic procedures rather than being mpMRI-guided, which may affect the generalizability of our findings. While we have focused on easily obtainable clinical parameters, future iterations of our model will consider incorporating additional features and conducting external validations to enhance its accuracy and applicability across diverse populations. We acknowledge that the absence of comorbidity and race/ethnic data could introduce biases. These factors can play significant roles in health outcomes and model predictions. This study’s retrospective design introduces several potential biases that must be considered when interpreting the findings. First, measured biases include demographic and clinical variability within the patient cohort, such as differences in age, PSA levels, and prostate volume. These factors, although statistically controlled, may still influence the model’s outcomes. Second, unmeasured biases could arise from the variability in mpMRI interpretation, as radiologic assessments may differ among clinicians, potentially affecting the consistency of PI-RADS scoring. Although cross-validation was applied to mitigate overfitting, the lack of external validation remains a limitation. The inclusion of patients with PI-RADS 1 and 2 scores, who underwent systematic transrectal biopsies, and those with PI-RADS 3, 4, and 5 scores, who underwent cognitive fusion biopsies, represents a potential source of variability. This discrepancy in biopsy protocols may have influenced the diagnostic outcomes and limits the direct comparability of results across patient groups. Future studies could address these potential sources of bias by adopting a prospective, multicenter approach, and employing standardized imaging protocols.

## 5. Conclusions

This study contributes to the evolving field by developing a robust ML model that integrates PSA values, mpMRI findings, and hematologic parameters, offering a holistic approach to predicting the necessity of prostate biopsy. By utilizing a diverse array of ML algorithms, including gradient boosting and ensemble methods, our research identifies the optimal predictive approach for this complex clinical challenge. Unlike previous studies, our model incorporates a comprehensive feature set that reflects routinely collected clinical data, including both imaging and laboratory findings. This integration enhances the model’s practical use, making it more adaptable to real-world clinical workflows. Despite its retrospective design and single-center data source, the model achieved strong predictive accuracy, with results suggesting its potential to reduce unnecessary biopsies and improve risk stratification.

With continued advancements in ML methodologies and the expansion of robust datasets, ML-based tools hold great promise in transforming PCa diagnostics. By integrating these tools into routine clinical practice, clinicians could achieve more personalized and evidence-based decision-making, reducing the burden of unnecessary procedures and improving patient outcomes. Ultimately, the successful implementation of such models has the potential to optimize healthcare resources, streamline diagnostic workflows, and enhance the overall quality of care in PCa management.

Future research should prioritize prospective, multicenter studies to validate the findings of this study and assess the model’s performance across diverse populations. Such efforts should aim to address current limitations, including the retrospective nature of data collection and the absence of external validation. Additionally, incorporating standardized imaging protocols and broader demographic representation would further enhance the model’s generalizability and clinical applicability.

## Figures and Tables

**Table 1:** The demographic and laboratory findings of the patients included in this study.

	Group 1 (*n* = 171)	Group 2 (*n* = 73)	*p*
Age (years) (median, min–max)	63 (36–76)	66 (45–78)	0.004 ^a^
Prostate Volume (cc) (median, min–max)	65 (15–220)	50 (15–216)	<0.001 ^a^
PI-RADS Score (*n*, %)			<0.001 ^b^
1	6 (3.5)	0 (0)	
2	69 (40.4)	15 (20.5)	
3	56 (32.7)	17 (23.3)	
4	31 (18.1)	19 (26)	
5	9 (5.3)	22 (30.1)	
Free PSA (ng/mL) (median, min–max)	1.46 (0.45–10.17)	1.38 (0.26–37.17)	0.444 ^a^
Total PSA (ng/mL) (median, min–max)	6.46 (1.36–24.78)	8.06 (4.08–1000)	0.001 ^a^
Free PSA/Total PSA (median, min–max)	0.223 (0.052–2.934)	0.154 (0.03–0.437)	<0.001 ^a^
PSA Density (PSA/Prostate Volume) (median, min–max)	0.102 (0.159–0.467)	0.163 (0.215–41.666)	<0.001 ^a^
Platelet (10^3^/µL) (median, min–max)	217 (88–559)	237 (114–481)	0.121 ^a^
Neutrophil (10^3^/µL) (median, min–max)	4.25 (1.34–13.82)	4.396 (1.74–10.29)	0.817 ^a^
Lymphocyte (10^3^/µL) (median, min–max)	2.1 (0.64–9.68)	2.27 (0.134–5.63)	0.166 ^a^
Monocyte (10^3^/µL) (median, min–max)	0.57 (0.06–2.98)	0.63 (0.35–1.15)	0.010 ^a^
NLR (median, min–max)	1.988 (0.554–13.021)	1.912 (0.611–7.687)	0.395 ^a^
MLR (median, min–max)	3.847 (0.617–29.166)	3.491 (0.186–8.35)	0.182 ^a^
PLR (median, min–max)	99.555 (21.694–319.428)	100.778 (30.373–1529.850)	0.898 ^a^
SII (median, min–max)	418.036 (116.497–2603.342)	432.117 (104.483–14,763.059)	0.838 ^a^

PI-RADS: Prostate Imaging Reporting and Data System, NLR: neutrophil-to-lymphocyte ratio. MLR: monocyte-to-lymphocyte ratio. PLR: platelet-to-lymphocyte ratio. SII: Systemic Immune Inflammation Index (Neutrophil × Platelet/Lymphocyte). ^a^ Mann–Whitney U, ^b^ Chi-Squared test.

**Table 2:** Prostate Cancer Prediction Results of Different Machine Learning Algorithms.

	Model Training Results					
	Test			Cross-Validation		
Model Name	ROC–AUC	Accuracy	Interval	ROC–AUC	Accuracy	Interval
AVG Blender	0.743	0.735	0.611–0.858	0.689	0.754	0.649–0.858
Logistic Regression	0.741	0.796	0.683–0.909	0.683	0.733	0.626–0.84
Extra Trees Classifier	0.808	0.755	0.635–0.876	0.72	0.708	0.597–0.818
LGBM Classifier	0.784	0.816	0.708–0.925	0.657	0.672	0.558–0.786
Random Forest Classifier	0.76	0.776	0.659–0.892	0.679	0.708	0.597–0.818
XGB Classifier	0.771	0.776	0.659–0.892	0.699	0.723	0.615–0.831

AVG Blender: Average Blender, LGBM: Light Gradient-Boosting Machine, XGB: eXtreme Gradient Boosting.

**Table 3:** Classifier Confusion Matrix.

	LGBM Classifier		
Prostate cancer	Yes	No	%
Yes	10	5	66.7 ^a^
No	4	30	88.2 ^b^

LGBM: Light Gradient-Boosting Machine. ^a^: Sensitivity, ^b^: Specificity.

## Data Availability

The data that support the findings of this study are available from the corresponding author, upon reasonable request.

## References

[1] Tarantino G., Crocetto F., Vito C.D., Martino R., Pandolfo S.D., Creta M., Aveta A., Buonerba C., Imbimbo C. (2021). Clinical factors affecting prostate-specific antigen levels in prostate cancer patients undergoing radical prostatectomy: A retrospective study. Future Sci. OA.

[2] EAU Guidelines EAU—EANM—ESTRO—ESUR—ISUP—SIOG Guidelines on Prostate Cancer. Presented at the EAU Annual Congress, Paris, France, 5–8 April 2024. https://uroweb.org/guidelines/prostate-cancer/.

[3] O’Shea A., Harisinghani M. (2022). PI-RADS: Multiparametric MRI in prostate cancer. Magn. Reson. Mater. Phys. Biol. Med..

[4] Schoots I.G., Padhani A.R. (2020). Personalizing prostate cancer diagnosis with multivariate risk prediction tools: How should prostate MRI be incorporated?. World J. Urol..

[5] Parekh S., Ratnani P., Falagario U., Lundon D., Kewlani D., Nasri J., Dovey Z., Stroumbakis D., Ranti D., Grauer R. (2022). The Mount Sinai Prebiopsy Risk Calculator for Predicting any Prostate Cancer and Clinically Significant Prostate Cancer: Development of a Risk Predictive Tool and Validation with Advanced Neural Networking, Prostate Magnetic Resonance Imaging Outcome Database, and European Randomized Study of Screening for Prostate Cancer Risk Calculator. Eur. Urol. Open Sci..

[6] Şahin E., Kefeli U., Zorlu Ş., Seyyar M., Ozkorkmaz Akdag M., Can Sanci P., Karakayali A., Ucuncu Kefeli A., Bakkal Temi Y., Cabuk D. (2023). Prognostic role of neutrophil-to-lymphocyte ratio, platelet-to-lymphocyte ratio, systemic immune-inflammation index, and pan-immune-inflammation value in metastatic castration-resistant prostate cancer patients who underwent 177Lu-PSMA-617. Medicine.

[7] Wang L., Li X., Liu M., Zhou H., Shao J. (2024). Association between monocyte-to-lymphocyte ratio and prostate cancer in the U.S. population: A population-based study. Front. Cell Dev. Biol..

[8] Luo Z., Wang W., Xiang L., Jin T. (2023). Association between the Systemic Immune-Inflammation Index and Prostate Cancer. Nutr. Cancer.

[9] Kaya C., Caliskan S., Sungur M., Aydın C. (2021). HALP score and albumin levels in men with prostate cancer and benign prostate hyperplasia. Int. J. Clin. Pract..

[10] Tătaru O.S., Vartolomei M.D., Rassweiler J.J., Virgil O., Lucarelli G., Porpiglia F., Amparore D., Manfredi M., Carrieri G., Falagario U. (2021). Artificial Intelligence and Machine Learning in Prostate Cancer Patient Management-Current Trends and Future Perspectives. Diagnostics.

[11] Aykaç A., Kaya C., Çelik Ö., Aydın M.E., Sungur M. (2024). The prediction of semen quality based on lifestyle behaviours by the machine learning based models. Reprod. Biol. Endocrinol..

[12] Bologna E., Ditonno F., Licari L.C., Franco A., Manfredi C., Mossack S., Pandolfo S.D., De Nunzio C., Simone G., Leonardo C. (2024). Tissue-Based Genomic Testing in Prostate Cancer: 10-Year Analysis of National Trends on the Use of Prolaris, Decipher, ProMark, and Oncotype DX. Clin. Pract..

[13] Suh J., Yoo S., Park J., Cho S.Y., Cho M.C., Son H., Jeong H. (2020). Development and validation of an explainable artificial intelligence-based decision-supporting tool for prostate biopsy. BJU Int..

[14] Yu S., Tao J., Dong B., Fan Y., Du H., Deng H., Cui J., Hong G., Zhang X. (2021). Development and head-to-head comparison of machine-learning models to identify patients requiring prostate biopsy. BMC Urol..

[15] Checcucci E., Rosati S., De Cillis S., Vagni M., Giordano N., Piana A., Granato S., Amparore D., De Luca S., Fiori C. (2022). Artificial intelligence for target prostate biopsy outcomes prediction the potential application of fuzzy logic. Prostate Cancer Prostatic Dis..

[16] Chiu P.K., Shen X., Wang G., Ho C.L., Leung C.H., Ng C.F., Choi K.S., Teoh J.Y.C. (2022). Enhancement of prostate cancer diagnosis by machine learning techniques: An algorithm development and validation study. Prostate Cancer Prostatic Dis..

[17] Checcucci E., Rosati S., De Cillis S., Giordano N., Volpi G., Granato S., Zamengo D., Verri P., Amparore D., De Luca S. (2023). Machine-Learning-Based Tool to Predict Target Prostate Biopsy Outcomes: An Internal Validation Study. J. Clin. Med..

[18] Chen S., Jian T., Chi C., Liang Y., Liang X., Yu Y., Jiang F., Lu J. (2022). Machine Learning-Based Models Enhance the Prediction of Prostate Cancer. Front. Oncol..

[19] Zhang H., Ji J., Liu Z., Lu H., Qian C., Wei C., Chen S., Lu W., Wang C., Xu H. (2023). Artificial intelligence for the diagnosis of clinically significant prostate cancer based on multimodal data: A multicenter study. BMC Med..

[20] Gu X., Gao X., Li X., Qi X., Ma M., Qin S., Yu H., Sun S., Zhou D., Wang W. (2016). Prognostic significance of neutrophil-to-lymphocyte ratio in prostate cancer: Evidence from 16,266 patients. Sci. Rep..

